# A Morally Permissible Moral Mistake? Reinterpreting a Thought Experiment as Proof of Concept

**DOI:** 10.1007/s11673-018-9845-x

**Published:** 2018-03-07

**Authors:** Nathan Emmerich, Bert Gordjin

**Affiliations:** 1 Institute of Ethics, Dublin City University, Dublin, Ireland; 2 School of History, Anthropology, Politics and Philosophy, Queen’s University Belfast, Belfast, UK

**Keywords:** Withdrawing, Withholding, Equivalence thesis, Morally permissible moral mistakes, Suberogatory acts

## Abstract

This paper takes the philosophical notion of suberogatory acts or morally permissible moral mistakes and, via a reinterpretation of a thought experiment from the medical ethics literature, offers an initial demonstration of their relevance to the field of medical ethics. That is, at least in regards to this case, we demonstrate that the concept of morally permissible moral mistakes has a bearing on medical decision-making. We therefore suggest that these concepts may have broader importance for the discourse on medical ethics and should receive fuller consideration by those working the field. The focus of the discussion we present is on a particular thought experiment originally presented by Sulmasy and Sugarman. Their case formed the basis of an exchange about the moral equivalence of withdrawing and withholding life-saving treatment. The analysis Sulmasy and Sugarman set out is significant because, contrary to common bioethical opinion, it implies that the difference between withdrawing and withholding life-saving treatment holds, rather than lacks, moral significance. Following a brief discussion of rejoinders to Sulmasy and Sugarman’s article, we present a constructive reinterpretation of the thought experiment, one that draws on the idea of suberogatory acts or “morally permissible moral mistakes.” Our analysis, or so we suggest, accounts for the differing moral intuitions that the case prompts. However, it also calls into question the degree to which this thought experiment can be thought of as illustrating the moral (non)equivalence of withdrawing and withholding life-saving treatment. Rather, we conclude that it primarily illuminates something about the ethical parameters of healthcare when family members, particularly parents, are involved in decision-making.

## Introduction

Whilst the notion of the supererogatory (Heyd 2016) has been considered in relation to certain bioethical issues—notably altruistic organ donation (Gerrand 1994, Wilkinson and Garrard 1996, 338) as well as in relation to medical practice and the profession itself (McKay 2002)—the same cannot be said of suberogatory acts (Driver 1992) or the closely related idea of morally permissible moral mistakes (Harman 2015a). These notions are, of course, supposed to parallel the idea of the supererogatory: acts that are subject to positive moral evaluations but are not considered obligatory. They encapsulate the notion that there may be some acts that are subject to negative moral evaluations but, nonetheless, are not morally verboten. As such, it is not obligatory to refrain from doing them and, furthermore, they need not be prevented or condemned outright.

This essay is intended as a “proof of concept,” that is, as a demonstration that, at least in the case discussed, the idea of a morally permissible moral mistake is relevant to medical ethics. Given the presumption that healthcare professionals ought to embody the highest of ethical ideals one might be inclined to think that we should not accommodate suberogatory acts. Whilst one might argue this point—and we offer a couple of further comments below—the case we discuss concerns the actions of the parents of two paediatric patients. It is they, and not the healthcare professionals involved, who make the morally permissible moral mistake, or so we argue. Thus, whilst our account is, we think, suggestive, further discussion of these concepts is required if we are to determine the ethical significance of the suberogatory or of morally permissible moral mistakes for the practice of medicine and healthcare. As a result, the extent of our substantive claim is that the idea of suberogatory acts, or of morally permissible moral mistakes, can provide insight into the ethical dimension of situations where healthcare professionals must deal with patient’s families rather than patients alone. Thus, they would seem to be important notions for both medical practice and medical ethics in such contexts.

Demonstrating the relevance of these concepts to this context involves revisiting a discussion about the moral equivalence of withdrawing and withholding of life-saving treatment. This discussion begins with a thought experiment that purports to show that, contrary to common bioethical opinion, the distinction between a treatment being withdrawn or withheld can have moral significance (Sulmasy and Sugarman 1994). Whilst many healthcare professionals clearly feel that some moral significance attaches to whether care is being withheld or withdrawn (Solomon et al. 1993; Dickenson 2000), in the discourse of both bioethics (Orentlicher 2001, chaps. 3 & 4) and professional guidelines (British Medical Association 2008), the common opinion is that there is no intrinsic moral distinction. The presumption that the distinction between withdrawing and withholding life-saving treatment carries no moral weight is so commonplace that it is, today, rarely debated. Rather it appears as a premise in arguments about the moral difference between withdrawing life-saving treatment and assisted suicide or the lack thereof (cf. Orentlicher 2001, chaps. 3 and 4).

The case presented by Sulmasy and Sugarman is, then, offered as a challenge to a view that is widely held by those working in the field. They term this view the Equivalence Thesis (ET) and state it as follows:

1. If it would have been morally permissible to have withheld a therapy (that has in fact already been started), then it is now morally permissible to withdraw that therapy; and:
2. If, in the future, it would be morally permissible to withdraw a therapy (that has in fact not yet been started), then it is now morally permissible to withhold that therapy (Sulmasy and Sugarman 1994, 218)

In order to account for their analysis of the case they set out, they introduce Nozick’s Principle of Original Acquisition of Holdings. Their claim is that the notion of prior acquisition provides a moral basis for distinguishing between withholding and withdrawing life-saving treatment. However, in his reply to Sulmasy and Sugarman, Harris (1994) suggests that the case should not be taken at face value. His claim is that they are comparing an ethical act—in this case the *decision* or the *decision-making process—*with one that is unethical. They are comparing a just decision to withhold life-saving medical treatment with an unjust decision to withdraw it. Given this point, there is a significant moral difference other than the bare fact of withdrawing and withholding. He therefore claims that the case does not provide a challenge to the ET. In an accompanying editorial, Gillon (1994) presents a similar view.

This paper interrogates the perspective presented by Sulmasy and Sugarman, as well as by Harris and Gillon. As intimated above, we suggest that a more nuanced understanding of the case can be achieved if we take up the notion of suberogatory acts (Driver 1992) and what Harman calls “morally permissible moral mistakes” (2015a). Similar to Harris and Gillon’s view, the picture we present calls into question the relevance of the case for the ET and whether the ET is a useful principle when it comes to the practicalities of withdrawing and withholding life-saving medical treatment. This additional point notwithstanding, if our analysis is convincing then, at minimum, we will have been successful in demonstrating that the idea of morally permissible moral mistakes has relevance to the medical ethics literature. Consequently, we hope others will join us in reflecting on these ideas further and consider if our ethical understanding of healthcare and medical practice could be improved by a broader application of these concepts. However, before pursuing such issues any further, we first offer a concise account of the way we understand the terms “suberogatory act” and “morally permissible moral mistakes.” We then set out the case presented by Sulmasy and Sugarman before briefly discussing Harris’ response and Gillon’s editorial. Finally, we offer a reconstructive analysis of the case in terms of morally permissible moral mistakes and discuss the implications of the picture we sketch.

## Suberogatory Acts and Morally Permissible Moral Mistakes

The notion of the suberogatory is conceptualized in parallel to the more common idea of supererogation. The idea of supererogation has been subject to continual and extensive analysis and discussion (Chisholm 1963; Heyd 1982; Mellema 1991; Postow 2005) since it was first put forward by Urmson in his seminal article “Saint and heroes” (1958). The same, however, cannot be said of suberogation. Whilst noting there is historical precedent for the category and that some modern philosophers have discussed it, Driver considers suberogation to be “almost entirely unknown” (1992, 286). Furthermore, it remains a controversial notion, and one might think the same of “morally permissible moral mistakes,” something recently introduced to the literature by Harman (2015a). As controversial categories of moral acts that have only recently been recognized, it is unsurprising that fully articulating them is a complex task. In order to simplify the challenge we face in doing so, we will treat the term “suberogation” and the phrase “morally permissible moral mistakes” as synonymous. However, one should note that Harman considers suberogatory acts to either partially overlap with her conception of morally permissible moral mistakes or to be a subclass of her notion (Harman 2015a, 370 fn.8). Whilst the limitations of space mean that we are unable to offer much in the way of broader discussion, this section sets out what we mean when we refer to suberogatory acts or morally permissible moral mistakes in this essay.

In Driver’s view, the idea of suberogation is a counterpart to that of supererogation.^1^ Acts of supererogation involve doing something that is morally good but not morally required. Suberogatory acts involve doing something that is morally bad but not morally forbidden. They are instances of poor behaviour which do not rise to the class of moral wrongs. The following examples are, at least arguably, instances of suberogation: failing to follow the social norms of polite discourse; failing to do (or return) a favour when the opportunity arises; and failing to donate a kidney to a sibling. Given that there is no *moral obligation* to be polite, to do (or even return) favours, or to donate a kidney to a sibling, any refusal to do these things is clearly not verboten, or a moral failure. Nevertheless, one might think that not doing such things represents some kind of moral error or mistake. Whilst not doing these things is not morally wrong or worthy of censure, it is nevertheless the case that not doing them is, in some way or in some sense, morally bad.^2^ It is such thinking that opens up space for the category of suberogatory acts or morally permissible moral mistakes; actions that are morally questionable but, nonetheless, are not forbidden and, in so far as that is the case, must therefore be considered permissible.

Harman’s conception of morally permissible moral mistakes is designed to capture a distinction similar to that of suberogation. In her view all morally wrong acts are moral mistakes but not all moral mistakes are morally wrong. These acts are *mere* moral mistakes, so called “because it is *something the agent should not do for moral reasons* and yet *it is morally permissible*” (Harman 2015a, 379 emphasis in original). Again, the idea is that we can have moral reasons not to do something but that it is nevertheless permissible to do it. As with Driver’s distinction between acts that are morally wrong and those that are morally bad, Harman’s construction of this point can strike one as questionable. However, consider the fact that whilst one might have moral reasons not to be rude to other people, it is nevertheless morally permissible to do so. Similarly, we may have moral reasons not to withhold favours from friends or kidneys from siblings. Nevertheless, it does seem morally permissible to withhold these things.

One might take Harman’s point as being concerned with substantive “demands” or “requirements” of morality or, at least, our moral philosophies. In this view, even if we take Harman to be concerned with ensuring that our theoretical accounts do not become so onerous as to be practically untenable, it would seem to have fundamental implications for the moral reality envisaged by moral philosophy. However, one might take Harman’s point as being concerned with the degree to which we might censure or, better, accommodate what appear to us to be the moral failings of others. From this perspective, the idea of morally permissible moral mistakes has less to do with our fundamental moral philosophies than it has to do with the moral realities we inhabit and the way we wish to structure them. As such, there is an underlying pragmatism to the idea of morally permissible moral mistakes. It is a name for acts that take place within a zone of collective moral uncertainty, something implied by a pragmatic adoption of moral pluralism. Consequently, Harman is raising questions concerning the degree to which we are required to tolerate or otherwise accommodate acts that we consider to be morally flawed or mistaken but that fall within this zone or sociocultural space.

This latter point is at its clearest in Harman’s discussion of moral vegetarianism and the meat eating of others (2015b). Regardless of the fact of the matter—if, indeed, there is any such thing—it is clear that moral vegetarians consider meat eating to be wrong. Given that a great many individuals who hold such views inhabit societies where meat eating is widespread there is, she says, “a puzzle about accommodation” (Harman 2015b): how far, if at all, should moral vegetarians accommodate the meat eating of others? One might think that moral vegetarians should not purchase and cook meat for their friends and family. But should they refuse to patronize retail outlets that also sell meat or any and all items that rely on animal products? Does this include products made by those who eat meat? Furthermore, should moral vegetarians refuse to go to restaurants that serve meat and, if they do, should they refuse to split the bill with their meat-eating friends? It is likely that a vegetarian meal will cost less than one that included meat. As a result, splitting the bill could be considered as subsidizing the meat eating of others as well as contributing to the profits of a business fundamentally built upon the sale of meat. Harman argues that such apparent contradictions can be understood by adopting the view that moral vegetarians consider meat eating to be a morally permissible moral mistake (Harman 2015a, 390, 2015b). In so doing, Harman is not claiming that this is, in fact, what moral vegetarians think. Rather, her point is that the concept of morally permissible moral mistakes provides a rationale for the way in which moral motivated vegetarians evidently accommodate meat eating.

As a result, one might take the notion of morally permissible moral mistakes as sketching out a category of action or behaviour that one might consider morally bad but, nevertheless, be prepared to tolerate or accommodate, at least to some degree. Of course, there might be limits to this tolerance—moral vegetarians might prefer not to form significant attachments with those who eat meat—and what might be accommodated in some contexts may not be accommodated in others. Nevertheless, it is clear that many of us regularly encounter instances of morally objectionable behaviour and, for the most part, we allow them to pass unchallenged and unremarked. Thus, even if one remains philosophically sceptical of suberogation or morally permissible moral mistakes as a theoretical feature of morality, it is clearly a feature or the moral reality we actually inhabit. For the purposes of this paper, then, suberogatory acts and morally permissible moral mistakes are morally questionable actions that, nevertheless, may require us to tolerate or accommodate them, at least in some cases.

## Sulmasy and Sugarman’s Case

The thought experiment set out by Sulmasy and Sugarman (1994) involves a pair of infant twins, Prima and Secunda. Both are suffering from carbon monoxide poisoning and, clinically speaking, are in exactly the same position. Without treatment both will die. There is, however, an available treatment: artificial ventilation. Unfortunately, the hospital only has one respirator. Thus, only one infant can receive the life-saving treatment they require. Having no other basis for their decision, the healthcare professionals caring for the twins decide to proceed via a random—which is to say *arbitrary*—allocation by, in essence, flipping a coin.^3^ Fortune favours Prima, meaning that she will receive treatment and will likely survive. Treatment will therefore be withheld from Secunda, and she will almost certainly die.

Just after this course of action has been initiated, the parents of Prima and Secunda arrive. Being properly apprised of the facts, their view is that, because she cries less than Prima, Secunda should be treated. Whilst treatment of Prima has already commenced, it has not yet had any effect. Therefore, or so Sulmasy and Sugarman stipulate, the clinical status of the infant twins remains identical. This will, of course, rapidly change. Sulmasy and Sugarman nevertheless maintain that, even if only for a very short period, the clinical status of the twins remains identical immediately after commencing treatment. Given their thought experiment and its stipulations, the implications of the ET are such that if, as seems to be the case, it was morally permissible to withhold treatment from Secunda and treat Prima then, as nothing of moral relevance has changed, it should now be permissible to withdraw treatment from Prima and treat Secunda. Sulmasy and Sugarman nevertheless suggest that the parents’ request should be rejected. They therefore argue that this case provides a counter example to the ET. Sulmasy and Sugarman conclude that the withholding and withdrawing of life-saving treatment is not always morally equivalent and that the ET is false, at least in some cases.

In order to justify the nonwithdrawal of treatment from Prima, Sulmasy and Sugarman (1994) lay claim to Nozick’s (1975) Principle of Original Acquisition of Holdings. They argue that this principle suggests the commencement of treatment creates a *prima facie* moral entitlement to the continuation and maintenance of that treatment or, at least, to not having it arbitrarily discontinued. This claim is not, however, inalienable and, in some cases, it may be preferable to withdraw care from one patient rather than withhold it from another, as when reverse triage is practiced, and treatment is withdrawn from patients who has a significantly lower chance of survival than those who would die if left untreated. Nevertheless, Sulmasy and Sugarman conclude that “the Equivalence Thesis is not a universal law of bioethics” (1994, 221). They claim that, because Nozick’s principle can be applied to all those who are already receiving treatment, there is a morally relevant difference between withdrawing and withholding life-saving treatment. If their account is correct, many bioethicists, and certain professional guidelines, are labouring under a significant misapprehension.

## Gillon’s Editorial and Harris’ Response

Sulmasy and Sugarman’s position is predicated on the view that had the parents been present at an earlier point in time, it would have been acceptable to act on their wishes, rather than on the basis of a random allocation. As Gillon (1994) points out, Sulmasy and Sugarman are not explicit about this point and, in his rejoinder, Harris (1994) calls it into question. Harris suggests that, regardless of when they arrive on the scene, those treating Prima and Secunda should not heed the wishes of the parents, claiming that the parents are “are not entitled to make an unjust choice” (Harris 1994, 223). His view is, then, that the parents’ decisions is morally flawed, and because of this, treating one twin rather than the other on the basis of the parents’ subjective preferences is unjust and should be rejected. Given this view, we might follow Gillon, who is editorializing against the backdrop of Harris’ comments, and think a justly made decision should not be overturned or, at least, not without “sound moral reasons for doing so” (Gillon 1994, 204).

However, say that the parents of Prima and Secunda were present in the hospital from the beginning, that they expressed their preference for Secunda to receive treatment and that, in the absence of any other motivation, a junior doctor elected to proceed on that basis. If a senior doctor^4^ subsequently arrived, would it be acceptable for this decision to be revisited? If a morally flawed decision has been made and followed, ought it be retaken in a morally just manner by, say, randomly (re)allocating the scarce resource? Harris’ account seems to suggest that doing so would be acceptable and he may even take the view that, at least until the clinical condition of Prima and Secunda diverges, there is a moral imperative to revisit it. In contrast, Sulmasy and Sugarman’s account would require some justification to overcome Secunda’s entitlement to continuation of her treatment. Depending on the weight they attach to “prior acquisition,” it may mean maintaining the original decision regardless of the “unjust” way in which it was made.

Both Sulmasy and Sugarman and Gillon and Harris, think that withdrawing treatment on the basis of the parents’ subjective preference would not be the right thing to do. We concur with this view. However, we do not do so in virtue of the principle of original acquisition, rather we agree with Gillon and Harris; the decision is unjust, and it need not be respected when it overturns a prior, justly made, decision. However, unlike Harris and Gillon, we think that, in the absence of a prior justly made decision, the subjective preference of the parents may be accommodated and allowed to motivate an initial allocation of resources. The parents’ decision certainly strikes us suberogatory. Nevertheless, it can be considered a morally permissible moral mistake.

## A Morally Permissible Moral Mistake?

Given our comments, we think it is possible to defend at least part of Sulmasy and Sugarman’s view—that is, the view that it would have been morally acceptable to act in accordance with the parents’ wishes if they had been present earlier—along the following lines. From the point of view of the healthcare professionals concerned, there is no basis for treating either Prima or Secunda. Only one life can be saved, and both have an equal claim. As a result, an *essentially arbitrary* decision must be made. This can either be done as a matter of random allocation, or it can be turned over to the parents. The thought experiment at hand defines the parents’ decision as subjective and, as a result, might be understood as bordering on the capricious. Indeed, some parents may refuse to make this choice, and some might consider it unethical for healthcare professionals to burden them with such a decision. Nevertheless, if the parents express such preferences then it may be that, from the point of view of healthcare professionals, proceeding on this basis is no more or less arbitrary than the random allocation of resources. In short, whilst we may think that the parents’ decision represents a moral mistake, having been asked to make it would seem morally permissible for them to make it in a morally flawed, or suberogatory, manner. Perhaps they should randomly —which is to say non-subjectively or arbitrarily, from their point of view—allocate treatment to one or other of their children. However, even if we think that they ought to do so, that does not necessarily mean that we should intervene if they do not. In this instance, it would be acceptable for healthcare professionals to accommodate the parents’ morally flawed decision.

When considering the suggestion that the parents ought to randomly allocate treatment to one or other of their children, one might even come to think that there is something ethically troubling about such an approach. Whether by flipping a coin or by some equivalent method, asking parents to make an arbitrary decision regarding their children’s lives would not seem to be a particularly ethical course of action. Of course, regardless of how such a decision is to be made, we could say the same about asking any parents to prioritize the treatment of one child over another. Furthermore, if one thinks that an arbitrary or random allocation of resources is, precisely, one way to avoid making a decision, there seems no need to ask the parents to decide in the first place. Nevertheless, asking them to acquiesce to such an approach to “decision-making” also seems problematic. At the same time, excluding them from such a decision would also seem troubling. At best, the situation is non-ideal, and there does not seem to be an entirely proper way to proceed.

In this context, then, even though we might regard the parents’ decision to subjectively favour one twin over the other as a suberogatory act or a morally permissible moral mistake, no other course of action seems entirely unobjectionable. As it seems to us that there are ethical concerns with all of the courses of action available, accommodating a suberogatory decision made by the parents would not, in itself, be unethical. Indeed, if the choice is between accepting the decision, and proceeding despite it being made in a flawed manner, or rejecting it, meaning that it must be retaken via random allocation—an act that will implicitly question the moral status of the original decision and, therefore, those that made it—then proceeding on the basis of a morally permissible moral mistake may be the morally best (or least worst) way forward. It is worth noting that, were the decision to be retaken, there would be a 50 per cent chance that the same twin will receive the treatment and, therefore, a 50 per cent chance that, excepting the damage done to the relationship between the parents and the healthcare professionals, nothing will change as a result of retaking the decision. Whilst this latter point might be considered by some as carrying little ethical weight it may, nevertheless, be accorded practical moral significance by those who must reject parental decision-making in order to arbitrarily decide the fate of infant twins on the flip of a coin. Even if their decision in this matter is unjust, it seems permissible to act on or accommodate the parents’ subjective preferences. The decision is made in a suberogatory manner, but it is a morally permissible moral mistake and, as such, healthcare professionals are not morally prohibited from following it.

## Implications for the Equivalence Thesis

Given our analytic reconstruction of Sulmasy and Sugarman’s thought experiment, one might reconsider its relevance for the ET. In our account of the case, it is morally permissible for healthcare professionals to withhold treatment on the basis of the parents’ suberogatory decision but, nonetheless, we do not think it is morally permissible to withdraw treatment on that same basis. This would seem to undermine the ET. However, if our account is taken as reflecting something about when it is and is not acceptable for healthcare professionals to act in accordance with morally permissible moral mistakes, then perhaps it should not be taken as informing the ET.^5^

Our view is that Sulmasy and Sugarman’s thought experiment can be understood as follows. The parents’ request to stop treating Prima and start treating Secunda can and should be rejected on the basis that a just decision has already been taken, and it should not be revised in an unjust manner. However, were the parents to be present at an earlier point in time, then it would be acceptable, if less than ideal, for Secunda to be treated in accordance with their less-than-just wishes. Making the decision in this way might be a moral mistake, but given that there is no morally relevant way to distinguish between deciding in one way or the other, and given that the parents’ preference can be considered as being no less arbitrary than the flip of a coin, then it seems morally permissible for the parents to decide, even if they do so in a suberogatory manner.

Such thinking might be consistent with Sulmasy and Sugarman’s suggestion that there is a moral distinction between withdrawing and withholding life-saving treatment. Our analysis indicates that it is permissible to withhold treatment on the basis of a suberogatory act, but that the same act should not be allowed to motivate the withdrawal of care. Alternatively, because Sulmasy and Sugarman’s discussion does not attend to the way in which the decisions are being made, we might agree with Harris’ view that the cases are not as comparable as Sulmasy and Sugarman suggest: one involves acting in accordance with a decision-making process that is just (random allocation) whilst the other involves acting in accordance with a decision-making process that is unjust (the subjective preference of the parents). Once this is seen as an important factor in the case, then what was previously accommodated as a morally permissible moral mistake may, if and when it overturns the just allocation of resources, be seen as a morally objectionable request. If so, then we have uncovered a heretofore-unrecognized moral difference between the instances of withdrawing and withholding in Sulmasy and Sugarman’s thought experiment.

This difference is, of course, related to the points made by Harris and Gillon. Nevertheless, it is more nuanced than their account would indicate. At least in some cases, it may be acceptable to accommodate morally suboptimal decision-making processes. Rather than being unjust per se, such cases can be considered as suberogatory acts or morally permissible moral mistakes. Such acts are certainly less than ideal and, as we have suggested, it may be that the same or similar decisions should be accommodated in some cases but not in others. Nevertheless, in the absence of a prior, justly made, decision, we might tolerate a morally malformed decision; we might not insist on it being retaken in a just manner. This could be taken as indicating the realities of clinical practice are such that the ET is a rather blunt tool and one that does not offer much in the way of insight into the moral landscape of withdrawing and withholding life-saving medical treatment. Thus, whilst our analysis of this case does not present a challenge to the ET—the relevant iterations of the case differ in a morally significant, if nuanced, manner—it contributes to our sense that the ET does not constructively shape the debate on withdrawing and withholding life-saving medical treatment.

## Implications for Dealing with Family Members

Healthcare professionals are often placed in positions that are less than ideal. In such contexts, they must do their best to discharge their responsibilities and do so in a manner that is ethical. Although it may be that the issue of conscientious objection can be fruitfully explored through the notion of morally permissible moral mistakes, this essay does not propose to incorporate such ideas into professional medical ethics; our intuition is that, with the possible exception of codifying guidelines for matters of contentious objection, professional guidelines should not detail a set of “moral mistakes” that are acceptable for healthcare professionals to make. Consequently, the issue of morally permissible moral mistakes is likely to be of primary interest insofar as the often less-than-ideal circumstances encountered by healthcare professionals may include instances where other people may make moral mistakes and where it might be permissible for them to do so. This includes making decisions in a suberogatory manner.

As a result, the primary question we should consider concerns when, and under what circumstances, might healthcare professionals be justified in rejecting morally permissible moral mistakes and when might they be accommodated? In cases where patients are making decisions for themselves, this seems relatively unproblematic. Whilst we do not accept the will of patients whose decision-making is *disordered* in some way, it seems clear that we tolerate, and even respect, the wishes of patients whose decision-making appears ill judged or misguided. The sovereignty we are entitled to exercise over ourselves—our autonomy— means that patients are generally free to make moral mistakes when it comes to making treatment decisions. There may, of course, be questions about how the patient’s decision impacts on others and any moral significance this might have. Nevertheless, our sociopolitical context is such that individuals have a great deal of latitude when it comes to making decisions for and about ourselves. Therefore, in such cases, there seems significant scope for morally flawed decisions to be considered permissible. However, when others become involved in making decisions for patients, healthcare professionals must more closely examine whether or not particular moral mistakes are permissible; they must consider if something is indeed a moral mistake or whether it is a moral wrong. The question posed by the phenomenon of suberogatory acts is, then, a matter of when we are required to challenge, intervene, or otherwise prevent such moral mistakes from determining the course of action to be taken in relation to non-autonomous patients and when we should not.

It seems to us that the actions of parents who make decisions based on their subjective preference for one child over another fall into the category of a morally permissible moral mistake, at least in some contexts. Viewed in this way, we can understand why it is that healthcare professionals may find themselves acting in accordance with these suberogatory decisions rather than challenging them. Of course, they may raise some form of challenge, at least in some cases and particularly in the context of shared decision-making. Nevertheless, even after raising some form of objection, they may ultimately decide to accommodate what they consider to be a mere moral mistake. Equally, we can appreciate that when a suberogatory decision contradicts a pre-existing and justly made decision, some healthcare professionals might refuse to act upon them. As a myriad of media reports make clear, there is significant scope for conflict between healthcare professionals and family members regarding patient care.^6^ To suggest that family members are not subject to the same ethical standards as healthcare professionals is, of course, fairly commonplace. However, the point that healthcare professionals may be required to tolerate and even respect suberogatory decisions made by family members has not previously been examined. This may be directly relevant to a range of issues in end-of-life care. For example, whether or not family members ought to be able to prevent the retrieval of organs from an individual who is a registered organ donor has been the subject of some debate (Wilkinson 2007). In the context of the above discussion and Driver’s (1992) comments about directed living donation mentioned above, we might think that, given it represents a moral failure to respect the wishes of the deceased registered organ donor, any family veto of post-mortem donation is an example of a morally permissible moral mistake. Indeed, we might think that it is a moral mistake to refuse to consent to post-mortem organ donation in general. Nevertheless, one might think that such a mistake ought to be considered morally permissible and that we ought not disregard them, as some have argued (Harris 2003).

Such thinking might be extended to cases where family members request inappropriate treatment for dying patients. Examples include CPR, continued life support, or the aggressive treatment of infections, such as pneumonia. Even if the healthcare professionals concerned would not recommend such treatment, and may even consider it unethical to do so, it may not be unethical to provide it given family requests. Furthermore, in some cases, we may be justified in revisiting decisions that have already been taken. Family members cannot, of course, compel healthcare professionals to provide inappropriate care. Nevertheless, there is a significant “grey area” that provides for uncertainty. In this context, healthcare professionals do not autonomously decide what to do, and a decision-making process may include family members. In pursuing a shared approach to decision-making, we should recognize that we have opened the door to the involvement of morally permissible moral mistakes in healthcare. As a result, healthcare professionals may need to accommodate decisions that are moral mistakes but are, nevertheless, permissible.

Consider the following example. It may be a moral mistake for the family of a frail and incompetent ninety-year-old to request CPR for their elderly relative. Nevertheless, it may be morally permissible for them to make such a mistake and for healthcare professionals to provide CPR on that basis. This should not, of course, mean that those providing care should abandon any involvement in such decisions. Healthcare professionals should, of course, engage with families, present their views, and share in the decision-making process. However, in cases where an impasse has been reached, healthcare professionals may need to tolerate or otherwise accommodate decisions that they feel are moral mistakes and, as a result, provide CPR that is unlikely to succeed or, pace our discussion, treat a child on the basis of the parents’ subjective preferences.

## Conclusion

Our view of Sulmasy and Sugarman’s case is that it is a morally permissible moral mistake for parental decisions to be predicated on a subjective preference for one child over another. Where this preference is expressed prior to any other justly made decision being taken, healthcare professionals might accommodate this preference and proceed accordingly. However, where this preference is expressed following a just decision-making process, they may legitimately ignore it. As such our analysis of the case does not pronounce any clear judgement on the ET; it falls foul of the ET’s *ceteris paribus* clause. All other things are not equal as there is an ethically significant difference between the instances of withholding and withdrawing life-saving treatment being compared. In the former instance, the decision is a morally permissible moral mistake. As a result, it may be accommodated without further error. In the latter instance, the parents’ decision would revise a justly made allocation of resources; accommodating it would involve an additional—and impermissible—moral mistake the part of healthcare professionals. However, whilst we cannot support Sulmasy and Sugarman’s approach to denying the validity of the ET, we feel our analysis contributes to a growing sense that viewing withholding and withdrawing as morally equivalent is a relatively blunt perspective on the matters at hand.

In addition to its implications for the ET, our analysis illuminates something that has, as yet, gone unremarked in the medical ethics literature. This is the notion that there is a class of actions, including decisions, that falls into the category of morally permissible moral mistakes. Healthcare professionals may encounter such suberogatory acts when dealing with family members. The question that must now be addressed is whether or not we can reliably identify such cases and how healthcare professionals ought to respond when they do so. In her analysis of whether, and to what degree, vegetarians ought to accommodate the meat eating of their non-vegetarian friends, Harman represents the concept of morally permissible moral mistakes as having explanatory power; it allows us to understand the common behaviour of vegetarians with regard to meat eating and meat eaters. However, we take the notion as having some degree of normative significance. Harman’s account does not just explain the behaviour of vegetarians but, now that it has been presented, may be taken as guiding future behaviour. Of course, there remains room for further argumentation and reflection as well as for individual differences; some vegetarians may accommodate what they see as the morally permissible moral mistake of meat eating to greater or lesser degrees. As there is no broad class of morally permissible but morally mistaken actions that patients often undertake, we do not present an argument to the effect that healthcare professionals ought to accommodate some specific behaviours. Nevertheless, if and when healthcare professionals find themselves concerned about the choices of patients, reflecting on whether or not the action falls into the category of morally permissible moral mistakes or if it is morally impermissible for them to act in this way, could offer normative guidance on how they might respond.

## Footnotes

1 Technically, suberogatory acts are a counterpart to a certain sort of supererogatory acts, what we might call non-heroic supererogatory acts. It is commonly thought that supererogation involves a great deal of self-sacrifice. This is not the case. Supererogatory acts are, simply, those that are morally good, whilst also being not morally required. Thus, whilst some supererogatory acts are highly self-sacrificing—giving all one’s money to charity or sacrificing one’s life to save that of others, for example—some instances of supererogation are not so costly. Examples include being polite (obeying social norms), sending unexpected gifts, or assisting others in some relatively minor way.

2 As Driver notes, using the term “morally bad” as distinct from “morally wrong” is, at best, awkward. However, Driver is attempting to capture an often-unrecognized category of acts: those that “are deserving of negative evaluation, without being actually wrong” (Driver 1992, 286) where those that are actually wrong are “impermissible” whilst those that are merely subject to negative evaluation are “permissible” or, at least, subject to tolerance and accommodation.

3 The image of flipping a coin is the very definition of random allocation. However, in all likelihood, were an act of random allocation to actually take place in practice, one of the healthcare professionals concerned would simply select one patient to receive treatment. Lacking any relevant basis for making a decision—and in the absence of any factor likely to invoke unconscious bias—any such selection will be sufficiently random or arbitrary.

4 Of course, from an ethical point of view, it should not matter if the initial decision was taken by a junior doctor and subsequently challenged by a senior doctor. However, for the sake of convenience, we rely on the fact of medical hierarchy to bolster our narrative.

5 It is worth being explicit at this point. Whilst the principle of prior acquisition introduces a moral difference between the two iterations of the thought experiment Sulmasy and Sugarman consider, this does not mean their comparison cannot inform the ET. This is because the difference is directly linked to the matter of withdrawing and withholding life-saving treatment. When we withdraw treatment, we withdraw what was previously acquired; when we withhold treatment, we withhold something that that has not yet been acquired.

6 At the time of writing, the most pertinant recent case is that of Charlie Gard, which generated a great deal of media coverage. As part of that coverage, The Guardian reported that the English courts had dealt with ten similar cases in the first six months of 2017 (Doward and Robertson 2017).
