# Correction to: A Morally Permissible Moral Mistake? Reinterpreting a Thought Experiment as Proof of Concept

**DOI:** 10.1007/s11673-019-09905-5

**Published:** 2019-03-18

**Authors:** Nathan Emmerich, Bert Gordijn

**Affiliations:** 1 Institute of Ethics, Dublin City University, Dublin, Ireland; 2 School of History, Anthropology, Politics and Philosophy, Queen’s University Belfast, Belfast, UK

**Correction to: Bioethical Inquiry (2018) 15:269–278**

10.1007/s11673-018-9845-x

There was a spelling error in the second author’s last name in the original publication. The name is correct in this erratum.

